# Supplementary material for: TopoDoE: a design of experiment strategy for selection and refinement in ensembles of executable gene regulatory networks
Source: BMC Bioinformatics. 2024 Jul 19;25:245. doi: 10.1186/s12859-024-05855-x (PMC11264509; doi:10.1186/s12859-024-05855-x)
Supplement: Supplementary file 2 — Supplementary Material 2. [file 12859_2024_5855_MOESM2_ESM.pdf]

Supplementary material

short name	Ensembl gene ID	short name	Ensembl gene ID
AACS	ENSGALG00000002899	ABCG2	ENSGALG00000005792
ACSL6	ENSGALG00000006644	ACSS1	ENSGALG00000008632
ANGPTL4	ENSGALG00000000619	ARHGEF2	ENSGALG00000006840
BATF	ENSGALG00000010323	BCL11A	ENSGALG00000007866
BPI	ENSGALG00000006756	CD151	ENSGALG00000006856
CRIP2	ENSGALG00000026471	CTCF	ENSGALG00000001817
CYP51A1	ENSGALG00000009365	DHCR7	ENSGALG00000004106
DHCR24	ENSGALG00000010798	DPP7	ENSGALG00000009105
EGFR	ENSGALG00000012363	FAM208B	ENSGALG00000008171
FHL3	ENSGALG00000001516	FNIP1	ENSGALG00000017462
GAB1	ENSGALG00000009898	GSN	ENSGALG00000001446
HMGCS1	ENSGALG00000014862	HRAS1	ENSGALG00000006885
HSD17B7	ENSGALG00000002681	HSP90AA1	ENSGALG00000011351
LCP1	ENSGALG00000016986	LDHA	ENSGALG00000006300
MFSD2B	ENSGALG00000016498	MID2	ENSGALG00000003860
MVD	ENSGALG00000007267	NSDHL	ENSGALG00000007493
PIK3CG	ENSGALG00000008081	PLS1	ENSGALG00000002647
REXO2	ENSGALG00000007024	RSFR	ENSGALG00000027165
RUNX2	ENSGALG00000026484	sca2	ENSGALG00000016152
SCD	ENSGALG00000005739	SLC6A9	ENSGALG00000010098
SLC25A37	ENSGALG00000000378	SNX22	ENSGALG00000002206
SQLE	ENSGALG00000016331	STARD4	ENSGALG00000000241
SULF2	ENSGALG00000004593	TBC1D7	ENSGALG00000012731
TNFRSF21	ENSGALG00000016719	ENSGALG00010025565	ENSGALG00010025565
VDAC3	ENSGALG00000003755		

S1 Table List of gene names and corresponding Ensembl gene IDs in the 364 candidate GRNs.

Test hypothesis	Gene name	p-value	q-value
	STARD4	0.9931	0.9996
	SLC25A37	0.0000	0.0000*
	ANGPTL4	0.9685	0.9996
	GSN	0.9700	0.9996
	FHL3	0.9961	0.9996
	CTCF	0.5773	0.9996
	SNX22	0.0001	0.0011*
	PLS1	0.3583	0.9996
	HSD17B7	0.9973	0.9996
	AACS	0.7651	0.9996
	VDAC3	0.3665	0.9996
less	MID2	0.9924	0.9996
	DHCR7	0.9900	0.9996
	SULF2	0.9561	0.9996
	SCD	0.9961	0.9996
	ACSL6	0.0741	0.5558
	BPI	0.9984	0.9996
	ARHGEF2	0.9923	0.9996
	CD151	0.9971	0.9996
	HRAS1	0.8347	0.9996
	REXO2	0.8648	0.9996
	MVD	0.9975	0.9996
	NSDHL	0.9514	0.9996
	BCL11A	0.8422	0.9996
	PIK3CG	0.9889	0.9996

Test hypothesis	Gene name	p-value	q-value
	FAM208B	0.7315	0.9996
	ACSS1	0.9640	0.9996
	DPP7	0.9849	0.9996
	CYP51A1	0.9941	0.9996
	SLC6A9	0.0001	0.0011*
	ENSGALG00010025565	0.0000	0.0000*
	BATF	0.9496	0.9996
	DHCR24	0.9656	0.9996
	HSP90AA1	0.9419	0.9996
	EGFR	0.9956	0.9996
	TBC1D7	0.0525	0.4725
less	HMGCS1	0.9338	0.9996
	sca2	0.9994	0.9996
	SQLE	0.9979	0.9996
	MFSD2B	0.8349	0.9996
	TNFRSF21	0.8440	0.9996
	LCP1	0.9712	0.9996
	CRIP2	0.9996	0.9996
	RUNX2	0.9850	0.9996
	RSFR	0.9120	0.9996
	STARD4	0.0069	0.0248
	SLC25A37	1.0000	1.0000
	ANGPTL4	0.0315	0.0675
	GSN	0.0300	0.0675
	FHL3	0.0039	0.0195

Test hypothesis	Gene name	Gene Ensembl ID	p-value	q-value
	CTCF	0.4227	0.5141	
	SNX22	0.9999	1.0000	
	PLS1	0.6417	0.7404	
	HSD17B7	0.0027	0.0186	
	AACS	0.2349	0.3020	
	VDAC3	0.6335	0.7404	
	MID2	0.0076	0.0248	
	DHCR7	0.0100	0.0300	
	SULF2	0.0439	0.0823	
	SCD	0.0039	0.0195	
	ACSL6	0.9259	1.0000	
greater	BPI	0.0016	0.0186	
	ARHGEF2	0.0077	0.0248	
	CD151	0.0029	0.0186	
	HRAS1	0.1653	0.2188	
	REXO2	0.1352	0.2028	
	MVD	0.0025	0.0186	
	NSDHL	0.0486	0.0872	
	BCL11A	0.1578	0.2188	
	PIK3CG	0.0111	0.0312	
	FAM208B	0.2685	0.3356	
	ACSS1	0.0360	0.0704	
	DPP7	0.0151	0.0378	
	CYP51A1	0.0059	0.0241	
	SLC6A9	0.9999	1.0000	

Test hypothesis	Gene name	p-value	q-value
	ENSGALG00010025565	1.0000	1.0000
	BATF	0.0504	0.0872
	DHCR24	0.0344	0.0704
	HSP90AA1	0.0581	0.0968
	EGFR	0.0044	0.0198
	TBC1D7	0.9475	1.0000
greater	HMGCS1	0.0662	0.1064
	sca2	0.0006	0.0135
	SQLE	0.0021	0.0186
	MFSD2B	0.1651	0.2188
	TNFRSF21	0.1560	0.2188
	LCP1	0.0288	0.0675
	CRIP2	0.0004	0.0135
	RUNX2	0.0150	0.0378
	RSFR	0.0880	0.1366

S2 Table: **Differential expression of T2ECs after FNIP1's Knock-Out** Gene expression levels of the KO cells compared to the wild type cells using one-sided T-tests (the 'less' hypothesis means that genes are down-regulated in the KO condition). The obtained p-values are reported in column 'p-value' and corrected p-values for multiple testing are reported in column 'q-value'. Asterisks in the table indicate significant q-values (q-value < 0.01).

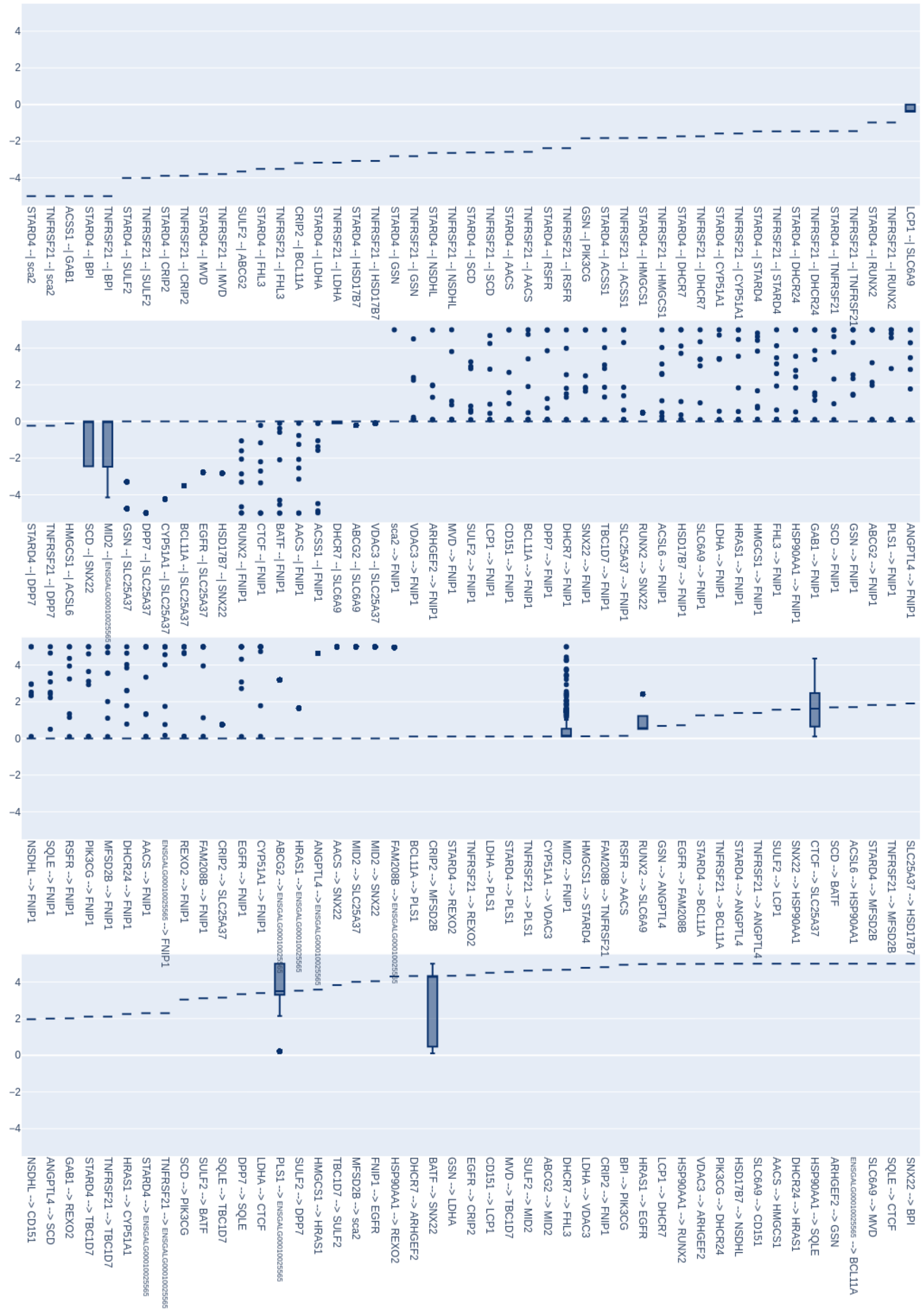

S1 Fig Boxplots of interaction values. Boxplots showing the distribution of interaction values for each stimulus-to-gene or gene-to-gene interaction which was non null in at least one of the 364 GRNs. Most interactions were identical (or close to being) in all GRNs, resulting in flat boxplots with few outliers.

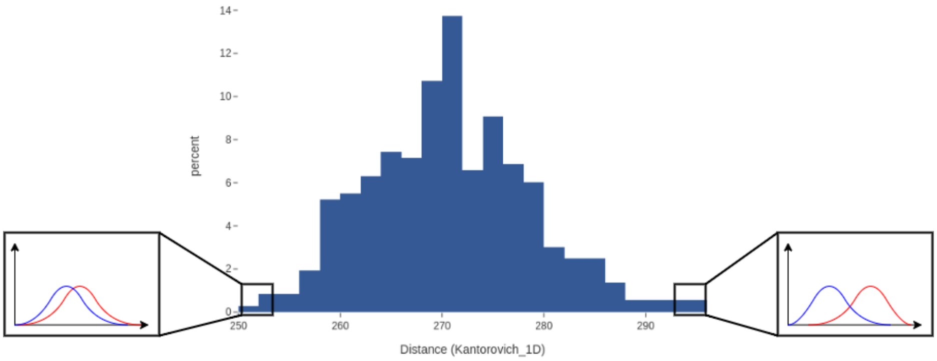

S2 Fig Distance to reference data. All 364 GRNs were simulated with the differentiation stimulus active for 72 hours and for each, mRNA expression data was recorded for 200 cells at time points 0h, 8h, 24h, 33h, 48h and 72h. Distributions of simulated expression data were compared to the reference experimental data (used in the inference process) using the $Kantorovich_{1D}$ distance. The histogram shows the distributions of the 364 obtained distances, where the leftmost bins hold the lowest distances (i.e. the distances of most similar distributions).

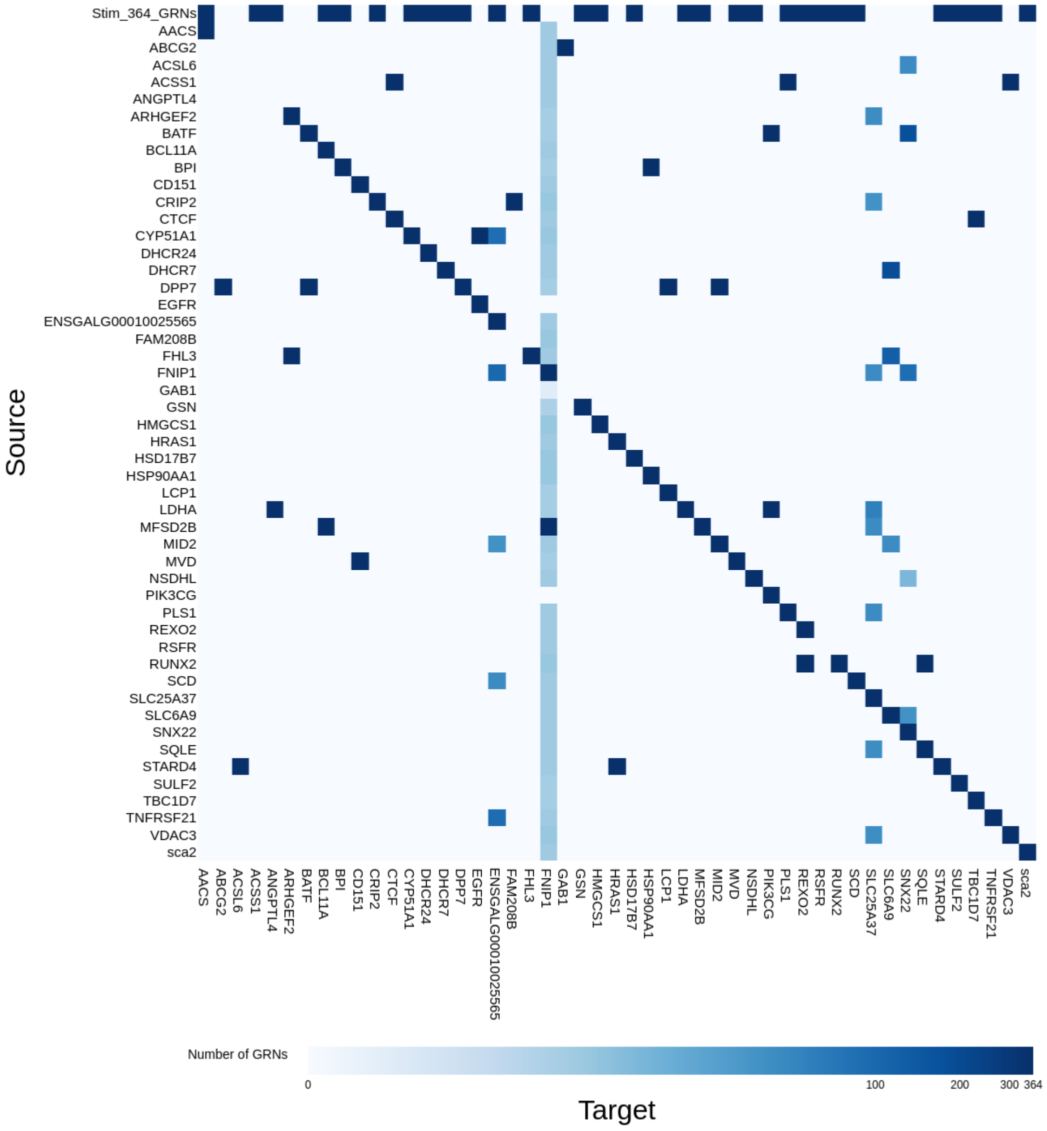

S3 Fig Matrix of the interaction consensus. Cells in the matrix indicate how many GRNs among the 364 candidates have an interaction between a gene (or stimulus) source (rows) and a gene target (columns). Values are color-coded on a log scale, where white indicates 0 and dark blue means 364.

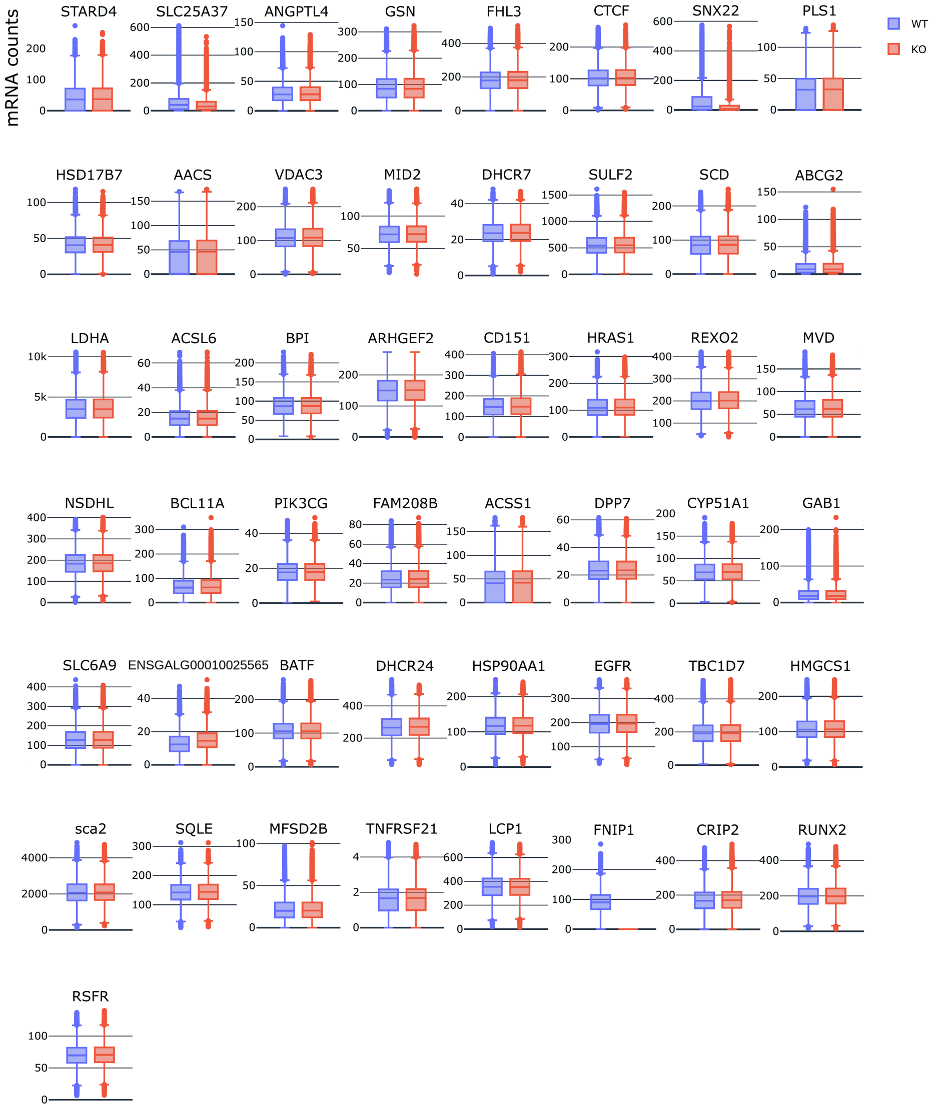

S4 Fig Simulations of *FNIP1*'s Knock-Out. Predicted expression variation for all 49 genes after *FNIP1*'s Knock-Out in all 364 GRNs. Wild type expression (no KO) is shown in **blue** while the KO condition is shown in **red**. Each box plot summarizes the expression level of 2000 simulated cells per each of the GRNs (728‘000 data points per box plot in total).

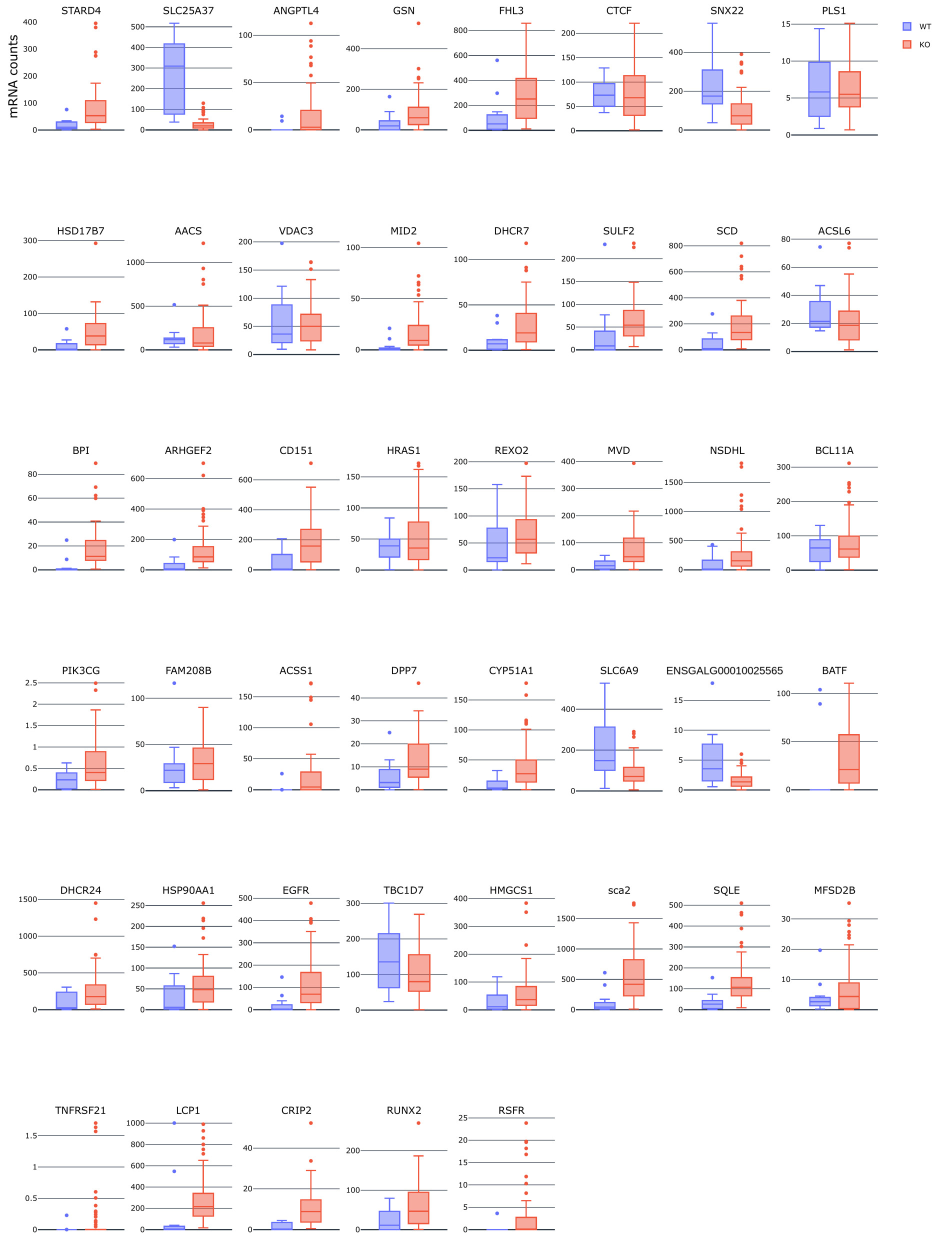

S5 Fig Experimental data of T2ECs after *FNIP1*'s Knock-Out. Observed expression variation for 45 genes (out of the 49 genes in the GRNs) after *FNIP1*'s Knock-Out in T2ECs. Missing genes are *ABCG2*, *LDHA*, *GAB1* and *FNIP1*. Wild type expression (no KO) is shown in **blue** while the KO condition is shown in **red**.

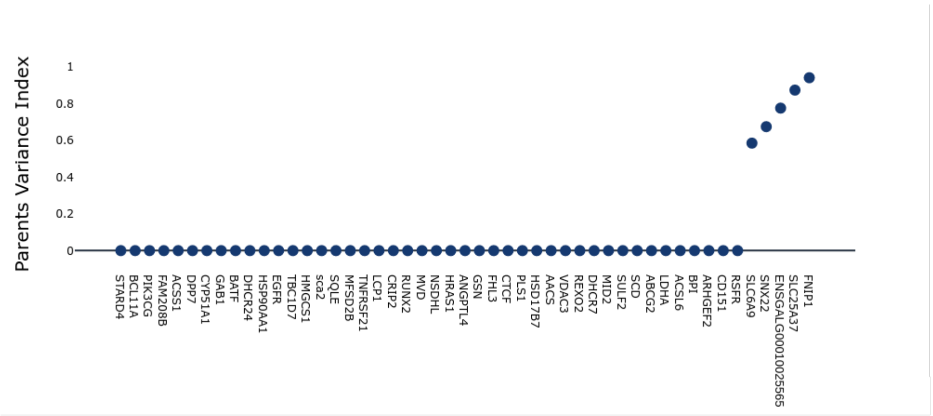

S6 Fig Ancestors Variance Index. The index of ancestors variance is computed as the sum of the variance of the interaction values, in all candidate GRNs, between a gene and its regulators. A high value indicates that a gene has interactions that vary a lot among all the candidate GRNs.
